# Quantitative ^99m^Tc-DPD-SPECT/CT assessment of cardiac amyloidosis

**DOI:** 10.1007/s12350-022-02960-3

**Published:** 2022-05-13

**Authors:** Lukas Kessler, Pedro Fragoso Costa, David Kersting, Walter Jentzen, Manuel Weber, Peter Lüdike, Alexander Carpinteiro, Sara Oubari, Tim Hagenacker, Andreas Thimm, Tienush Rassaf, Ken Herrmann, Maria Papathanasiou, Christoph Rischpler

**Affiliations:** 1 Department of Nuclear Medicine, University Hospital Essen, Hufelandstrasse 55, 45147 Essen, Germany; 2 Department of Cardiology and Vascular Medicine, West German Heart and Vascular Center, University Hospital Essen, Hufelandstrasse 55, 45147 Essen, Germany; 3 Department of Hematology and Stem Cell Transplantation, West German Tumor Center, University Hospital Essen, Hufelandstrasse 55, 45147 Essen, Germany; 4 Department of Molecular Biology, University of Duisburg-Essen, Hufelandstrasse 55, 45147 Essen, Germany; 5 Department of Neurology, University Hospital Essen, Hufelandstrasse 55, 45147 Essen, Germany

**Keywords:** Cardiac amyloidosis, ATTR, AL, imaging, SPECT, quantification, Perugini

## Abstract

**Introduction:**

Transthyretin (ATTR) amyloidosis is responsible for the majority of cardiac amyloidosis (CA) cases and can be reliably diagnosed with bone scintigraphy and the visual Perugini score. We aimed to implement a quantification method of cardiac amyloid deposits in patients with suspected cardiac amyloidosis and to compare performance to visual scoring.

**Methods and materials:**

136 patients received ^99m^Tc-DPD-bone scintigraphy including SPECT/CT of the thorax in case of suspicion of cardiac amyloidosis. Imaging phantom studies were performed to determine the scaling factor for standardized uptake value (SUV) quantification from SPECT/CT. Myocardial tracer uptake was quantified in a whole heart volume of interest.

**Results:**

Forty-five patients were diagnosed with CA. A strong relationship between cardiac SUVmax and Perugini score was found (Spearman *r* 0.75, *p* < 0.0001). Additionally, tracer uptake in bone decreased with increasing cardiac SUVmax and Perugini score (*p* < 0.0001). ROC analysis revealed good performance of the SUVmax for the detection of ATTR-CA with AUC of 0.96 ± 0.02 (*p* < 0.0001) with sensitivity 98.7% and specificity 87.2%.

**Conclusion:**

We demonstrate an accessible and accurate quantitative SPECT approach in CA. Quantitative assessment of the cardiac tracer uptake may improve diagnostic accuracy and risk classification. This method may enable monitoring and assessment of therapy response in patients with ATTR amyloidosis.

**Supplementary Information:**

The online version contains supplementary material available at 10.1007/s12350-022-02960-3.

## Introduction

Cardiac amyloidosis (CA) is an underdetected cause for heart failure which has significantly gained attention in the past years.^1,2^ Systemic amyloidosis is a disorder that leads to extracellular deposition of misfolded proteins affecting organ function. In the vast majority of cases light-chain (AL) or transthyretin (ATTR) amyloid deposits are responsible for CA, leading to systolic and diastolic dysfunction, hypertrophy, arrhythmias, conduction blocks, and heart failure. Cardiac involvement is the most significant prognostic factor in patients with amyloidosis.^3–7^ AL amyloidosis can be treated with different anti-plasma cell regimens.^8^ In case of hematological response^5^ organ responses with improving organ function are possible. In ATTR amyloidosis disease specific therapeutic approaches include transthyretin stabilizers or transthyretin gene silencers.

Until recently endomyocardial biopsy has been the “gold standard” for diagnosing all types of cardiac amyloidosis, but non-invasive strategies are emerging.^3,9,10^ It has been known for few years now that accumulation of bone-seeking radiopharmaceuticals like ^99m^Tc-3,3-diphosphono-1,2-propanodicarboxylic acid (^99m^Tc-DPD) have very high accuracy in diagnosing ATTR amyloidosis. In 2005, a visual scoring system known as the Perugini score was introduced.^11^ In 2016, a large multicenter study showed that substantial cardiac radiotracer uptake (Perugini score >1) on bone scintigraphy yields a specificity of 100% for cardiac ATTR amyloidosis when plasma cell disease is excluded.^3^ In light of these findings, new diagnostic algorithms were implemented, and bone scintigraphy is becoming increasingly important in the diagnostic and differentiation of cardiac amyloidosis often obviating the need for endomyocardial biopsy.^12^

Currently the visual interpretation and scoring of the myocardial uptake of ^99m^Tc-phosphate tracers based on Perugini is the standard in clinical practice. Nonetheless, there are certain limitations due to lack of reliable quantification and frequent neglection of SPECT/CT data.

Methods for quantitative SPECT have been emerging in the last years but “real-life” applications are still lacking. The previous unavailability of accurate SPECT reconstructions is being increasingly replaced by hybrid SPECT/CT systems, which can be setup for true quantification or are intrinsically capable of tracer quantification.^13^

Quantification of cardiac tracer uptake could enhance diagnostic capabilities of bone scintigraphy and allow assessment of disease progression and therapy response. So far only a few studies have investigated different approaches to (semi-)quantify tracer uptake in cardiac amyloidosis.^14–16^ A recent study showed similar or slightly better sensitivity and specificity for detection of transthyretin amyloidosis with semiquantitative parameters compared to visual scoring.^17^ First approaches for true quantitative SPECT/CT in CA have been reported using commercially available SPECT/CT quantification methods.^16,18^ These methods either rely on manually adjusted volume of interests or threshold based isocontours, which are prone to misinterpretation in patients with low or no cardiac tracer uptake.^16,18^ Further these methods are bound to commercially available software solutions and not easily transferable to other institutions. Despite that, their results showed the feasibility of this technique. The aim of this study was to implement an accessible, accurate and robust whole heart quantification method for cardiac amyloid deposits in patients with suspected cardiac amyloidosis and to compare results to visual Perugini scoring.

## Methods and materials

### Study population

**Figure 1:**
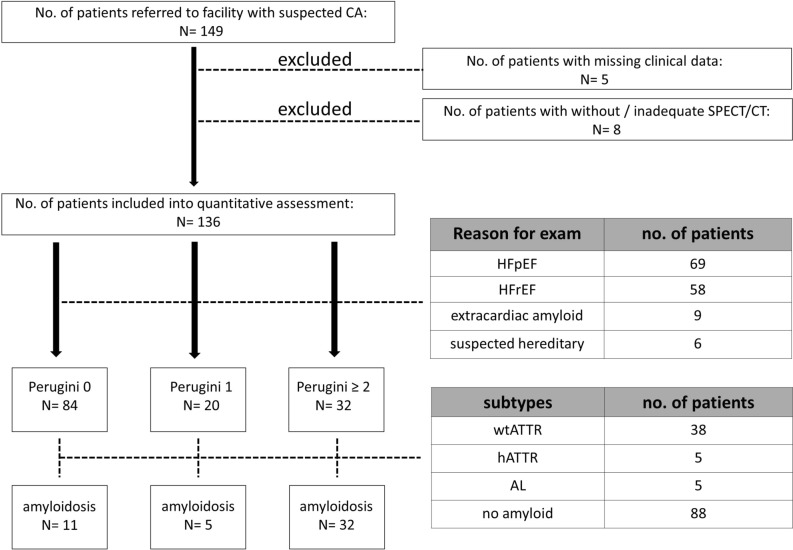
Patient selection. 149 patients were screened. Five patients were excluded due to missing clinical data (e.g., weight, accurate acquisition delay, diagnosis). Eight patients were excluded because of missing or insufficient SPECT/CT for quantitative assessment. HFpEF, heart failure with preserved ejection fraction; HFrEF, heart failure with reduced ejection fraction; wtATTR, wildtype transthyretin amyloidosis; hATTR, hereditary transthyretin amyloidosis; AL, light-chain amyloidosis

Between 10/2017 and 05/2020 149 patients with suspected CA were referred to the nuclear medicine facility. 136 patients were imaged with a ^99m^Tc-DPD scintigraphy including SPECT/CT of the thorax in the routine diagnostic workup of suspected CA (Figure 1) and were included in the quantitative assessment. CA was diagnosed according to the Gillmore Criteria^3^ by endomyocardial biopsy or bone scans and analysis of protein electrophoresis, serum free light-chain assay and immunofixation in urine and blood samples. All patients gave their written informed consent to the examination. Data were retrospectively analyzed and anonymously handled, in accordance with the Declaration of Helsinki. Ethics approval was granted by the ethics and human research committee of the University of Duisburg-Essen, Germany (20-9278-BO).

### Data acquisition and SPECT/CT reconstruction

All phantom and patient data were acquired on hybrid SPECT/CT systems (Symbia T2 and Symbia Intevo, Siemens Medical Solutions AG, Erlangen, Germany). The quantitative description of the tracer uptake in SPECT clinical images was implemented by a calibration step, which consisted of two controlled measurements of calibrated ^99m^Tc solutions. The first measurement was a planar measurement of a point source to define the system’s efficiency (cps/kBq) to 140 keV photons detection on the collimator/gamma camera system. Images were scatter corrected, applying a lower scatter window subtraction to the photopeak channel. Secondly, a cylindrical phantom 20 cm long and 20 cm in diameter was scanned in identical conditions of those in the clinical routine, 15 seconds per view in step-and-shoot mode, with a total of 64 views, zoom 1.0, noncircular orbit with body contour. A low-dose CT was performed immediately after emission acquisition. Patient and phantom SPECT data were reconstructed to a 128x128-pixel matrix, using the ordered subset expectation maximization iterative algorithm with 3D collimator modeling (Flash 3D), scatter correction was performed (as described above) and a smoothing Gaussian filter of 9mm FWHM was applied. All SPECT data were corrected for attenuation based on the low-dose CT. Original tomographic data were converted to radiotracer activity concentration applying the following relationship:

$$AC = \overline{{VOI}} _{{{\text{voxel}}}} \left[{t_{{{\text{acq}}}}  \cdot E \cdot V_{{{\text{voxel}}}}} \right]^{{ - 1}},$$

where AC is the activity concentration in (kBq/mL), ${\overline{VOI} }_{voxel}$ the mean count within the volume of interest, ${t}_{\text{acq}}$ the total SPECT measurement time in s, $E$ efficiency in cps/kBq and finally ${V}_{\text{voxel}}$ the voxel volume in the reconstructed SPECT matrix in mL.

### Image analysis

DICOM images were analyzed with Pmod v3.2 (Zurich, Switzerland). CT images were used to manually draw a freehand VOI around the whole heart. The whole heart was segmented because left ventricular myocardium cannot be reliably identified on non-contrast CT. Co-registration of SPECT and CT fusion images was confirmed and then the VOI was copied and pasted to the attenuation-corrected (AC) SPECT images. As references spherical VOIs of 1cm diameter were placed in the thoracic aorta (blood pool) and a vertebral bone without signs of osteoarthritis on CT or bone scintigraphy. VOI statistics was performed and the related count values (min, max, mean, standard deviation and total counts) were exported. Visual scoring of planar images was performed by two trained nuclear medicine physicians independently according to the previous described Perugini method.^11^

Standard uptake value (SUV) was calculated by the known equation:

SUV = $$\frac{{\text{voxel activity concentration }}\left(\frac{{\text{Bq}}}{\text{ml}}\right)*{\text{patient weight}}\left({\text{g}}\right)}{{\text{decay corrected injected activity}}(\text{Bq})}*1\frac{ml}{g}.$$

In addition, SUVmax values were normalized either to the background activity in the blood pool or to the tracer uptake of a vertebral bone as reference, both ongoing referred as

Myocardium-to-Blood pool ratio (MBR) or Myocardium-to-vertebral bone ratio (MVR).

### Statistical analysis

Data were statistically analyzed with GraphPad Prism 8.4.1. Continuous data are reported as mean ± standard deviation (SD). Correlation between visual Perugini scores and uptake values were evaluated by linear regression and the Spearman rho-method for nonparametric data. Kruskal–Wallis analysis of variance was used when comparing more than 2 groups as the omnibus test. Differences between nonparametric data were calculated pairwise by Mann–Whitney U tests. *p* value < 0.05 were considered as statistically significant. Contingency was analyzed by Fisher’s exact test. Confidence intervals were constructed using the Wilson score method. Diagnostic performance was analyzed by calculation of the area under the receiver operating characteristic curve.

## Results

### Study population

The study included 136 patients (89 male, 47 female) with a mean age of 76 ± 10 years. Patients were injected with a mean activity of ^99m^Tc-DPD of 523 ± 27 MBq (range 484-635). Late static images approximately 3h p.i. were used for analysis. Most patients was referred for ^99m^Tc-DPD scintigraphy because of heart failure with preserved ejection fraction (n = 69) or reduced ejection fraction (n = 58), nine patients were referred due to extracardiac amyloid deposits and six patients with suspected hereditary amyloidosis (Figure 1). Forty-five (33.1%) patients were diagnosed with CA (detailed in Supplementary Table 1). Of those, 36 patients were diagnosed with wtATTR (80 %), 4 with hATTR (8.8 %) and 5 with AL amyloidosis (11.1%) (Figure 1; Table 1). Planar images of bone scans were evaluated by two independent readers and Perugini score was reported. 84 patients with Perugini 0, 20 with Perugini 1 and 32 with Perugini 2 or higher (Figure 1; Table 1). There was no significant difference between demographic and imaging data among patients with different Perugini scores (Table 1). For 121 patients, echocardiography prior to ^99m^Tc-DPD scintigraphy was available (Table 1). Detailed parameters are shown in Table 1, of note patients with Perugini 3 showed significantly lower LVEF to Perugini 0 patients (*p* < 0.01) and higher LVMI to Perugini 0 (*p* < 0.001) and Perugini 1 patients (*p* < 0.05).

**Table 1:** Baseline patient characteristics and uptake values

	Grade 0 (*n* = 84)	Grade 1 (*n* = 20)	Grade 2 (*n* = 9)	Grade 3 (*n* = 23)	*p* value
*Demographic data*					
Male/Female	56 / 28	11 / 9	4 / 5	18 / 5	0.22
Age	76.0 ± 9.8	75.4 ± 13.6	75.3 ± 13.0	79.0 ± 7.9	0.60
Imaging					
Acquisition delay (min)	236.9 ± 50.6	218.9 ± 49.3	208.8 ± 62.8	231.5 ± 56.1	0.29
Injected activity (MBq)	518.5 ± 22.0	532.8 ± 36.2	536.6 ± 36.9	526.1 ± 26.4	0.051
Amyloid type					
wtATTR	4 (4.8)	5 (25.0)	7 (77.8)	22 (95.6)	**<0.0001**
hATTR	2 (2.4)	**0**	2 (22.2)	1 (4.4)	**<0.05**
AL	5 (5.9)	**0**	**0**	**0**	0.64
No amyloid	73 (86.9)	15 (75.0)	**0**	**0**	**<0.0001**
*Echocardiography*					
LVEF	51.6 ± 11.4	51.0 ± 8.9	51.7 ± 12.0	43.0 ± 10.9	**<0.01**
TAPSE	18.3 ± 5.3	19.4 ± 5.8	16.9 ± 5.4	15.4 ± 4.7	0.07
SPAP	44.4 ± 17.3	47.7± 16.1	41.8 ± 9.4	38.4 ± 12.0	0.36
LVMI	140.3 ± 52.8	147.1 ± 61.8	155.5 ± 25.5	197.6 ± 64.2	**<0.01**
IVSd	13.7 ± 4.1	13.1 ± 4.6	13.9 ± 3.0	17.2 ± 5.6	**<0.01**
LVEDD	48.9 ± 8.3	50.6 ± 8.1	47.1 ± 9.6	47.8 ± 9.9	0.49
PWd	12.1 ± 3.1	12.2 ± 4.7	14.9 ± 3.0	17.8 ± 9.3	**<0.001**
LAVI	63.1 ± 33.2	79.4 ± 39.9	47.4 ± 11.8	55.8 ± 18.1	0.11
e’	8.6 ± 3.9	9.1 ± 6.7	7.3 ± 2.1	5.9 ± 2.1	0.06
*SUVmax*					
Cardiac	2.5 ± 0.9	4.0 ± 1.8	15.2 ± 3.4	14.8 ± 4.2	**<0.0001**
Blood pool	1.7 ± 0.8	2.1 ± 0.6	1.9 ± 0.7	1.6 ± 0.5	**<0.01**
Vertebral	9.9 ± 3.1	9.7 ± 3.8	7.5 ± 2.6	6.0 ± 1.7	**<0.0001**

Values are n (%) or mean ± SD. Bold values are statistically significant. LVEF, left ventricular ejection fraction; TAPSE, tricuspid annular plane systolic excursion; SPAP, systolic pulmonary artery pressure; LVMI, left ventricular mass index; IVSd, interventricular septal thickness end diastole; LVEDD, left ventricular end-diastolic dimension; PWd, posterior wall thickness end diastole; LAVI, left atrial volume index

### Quantitative SPECT/CT

**Figure 2:**
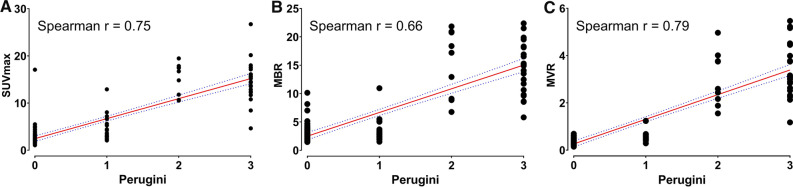
Scatter diagrams show a linear relationship between the visual Perugini score and tracer uptake parameters (**A**–**C**). Spearmans’ r shows strong correlation for all parameters

Quantitative SPECT/CT data showed consistent results compared to planar images. Myocardial SUVmax (*r* = 0.75, *p* < 0.0001; Figure 2A) and normalized SUVmax values showed a good linear relationship with Perugini score (MBR *r* = 0.69, *p* < 0.0001, Figure 2B; MVR r = 0.81, *p* < 0.0001; Fig. 2C).

**Figure 3:**
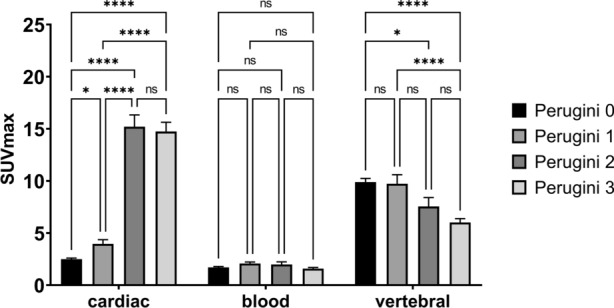
Quantitative tracer uptake was measured in a freehand VOI of the heart and spherical VOIs in the blood pool and a vertebral bone. Calculated SUVmax shows significantly increase tracer uptake in the heart with increasing Perugini score. Furthermore, a decrease of bone tracer uptake could be observed in patients with Perugini score 2 and 3. *p < 0.05; ****p < 0.0001

In line with the visual scoring the quantitative data revealed a steady increase in tracer uptake in the myocardium with a mean SUVmax of 2.5 ± 0.9 at Perugini 0 and mean SUVmax of 14.8 ± 4.2 at Perugini 3 (Table 1; Figure 3). Concurrently, a significant decrease in tracer activity in vertebral bone in patients with Perugini 3 compared to Perugini 0 and 1 (****p < 0.0001) and between Perugini 0 and Perugini 2 (*p < 0.05) (Figure. 3) was observed. Blood pool tracer activity quantified as SUVmean showed no significant change in the four groups. Similar results were seen with normalized SUV parameters (data not shown).

**Figure 4:**
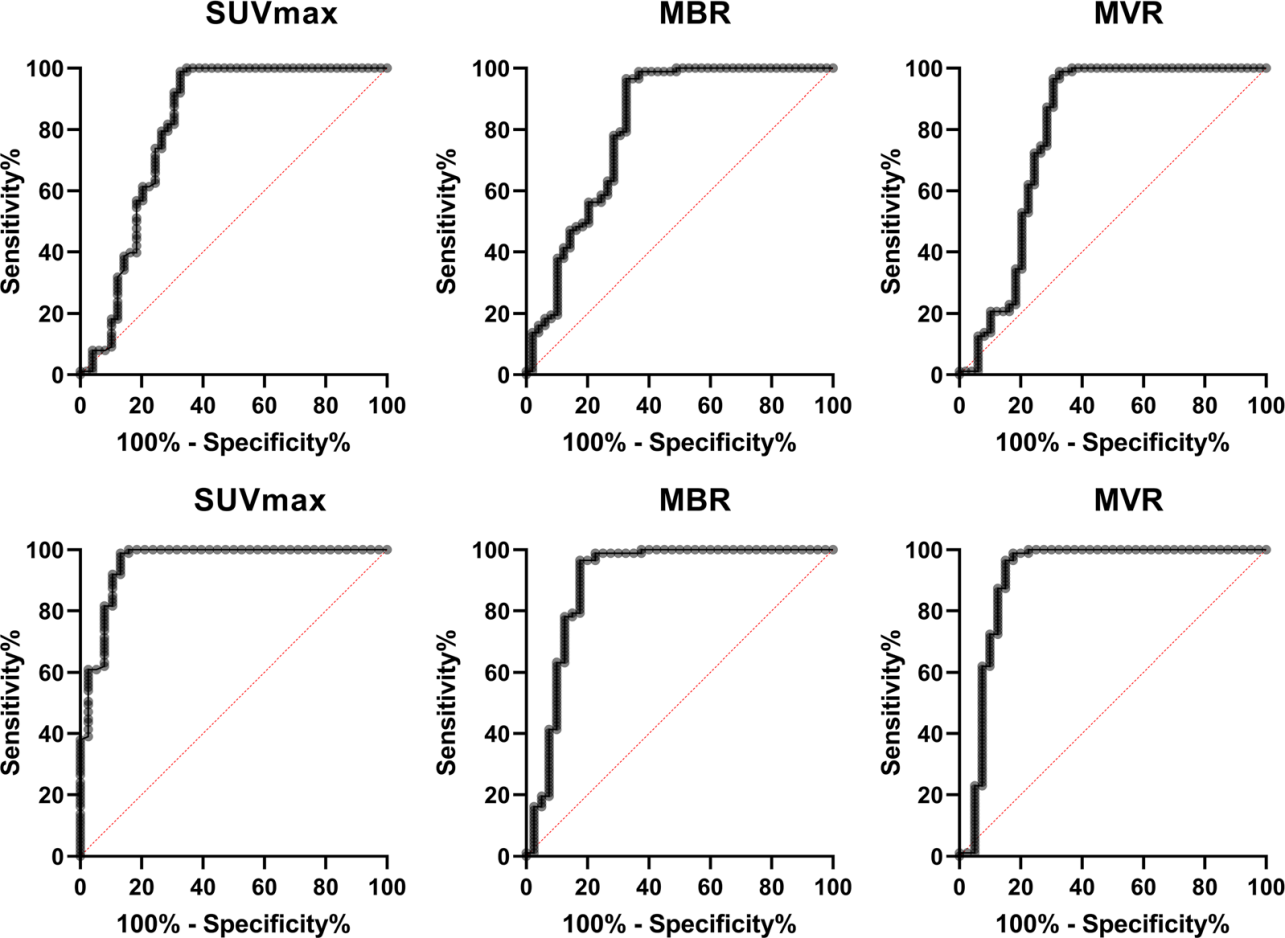
Receiver operating characteristics. ROC analysis of patients with any cardiac amyloidosis (**A**) or ATTR amyloidosis (**B**) vs. no amyloidosis show similar performance for SUVmax, MBR, MVR. All three methods show acceptable sensitivity and specificity, e.g., cardiac SUVmax with sensitivity 99% and specificity 87% at a cut-off SUVmax of 6.1 for the diagnosis of ATTR amyloidosis. MBR, myocardium-to-blood ratio; MVR, myocardium-to-vertebral bone ratio

Results of the ROC analysis are depicted in Figure 4 and Table 2**.** Patients with any type of CA (Figure 4A) and ATTR subtype (hATTR and wtATTR; Figure 4B) were compared to patients with no CA. All the established SUV quantities showed similar results, for convenience data will be focused on SUVmax. Area under the curve (AUC) for any CA was 0.81 ± 0.04 (****p < 0.0001) (Figure 4A) and 0.96 ± 0.02 (****p < 0.0001) for the ATTR type (Figure 4B). At a cut-off SUVmax > 6.1, there was acceptable sensitivity and specificity of 98.7% (95% CI 92.7-99.9%) and 87.2% (95% CI 73.3-94.4%) for cardiac ATTR amyloidosis (Table 2).

**Table 2:** Correlation and detection accuracy

	Cardiac SUVmax	MBR	MVR)
*Correlation*			
Spearman *r*	0.75	0.66	0.79
95% confidence interval	0.67 to 0.82	0.55 to 0.75	0.72 to 0.85
p-value	**<0.0001**	**<0.0001**	**<0.0001**
*ROC for ATTR amyloidosis*			
Area under curve	0.96	0.9	0.96
Std. Error	0.02	0.04	0.02
95% CI	0.93 to 1.00	0.81 to 0.98	0.91 to 1.00
p-value	**<0.0001**	**<0.0001**	**<0.0001**
*Sensitivity and Specificity for ATTR amyloidosis*			
Cut-off	< 6.1	< 8.3	< 0.66
Sensitivity%	98.7	98.7	98.7
95% CI	92.7% to 99.9%	92.8% to 99.9%	92.8% to 99.9%
Specificity%	87.2	78.8	84.9
95% CI	73.29% to 94.40%	62.25% to 89.32%	69.08% to 93.35%
Likelihood ratio	7.7	4.7	6.5

Spearman’s r correlation of cardiac uptake and Perugini Score. Normalized cardiac SUVmax by mean uptake in blood pool (MBR) or vertebral (MVR). Bold values are statistically significant (p<0.05). MBR, myocardium-to-blood ratio; MVR, myocardium-to-vertebral bone ratio

Further, diagnostic performance of SPECT/CT quantification at a cut-off of SUVmax >6.1 was compared to visual Perugini scoring (Table 3). In this cohort SPECT/CT quantification showed a higher sensitivity compared to Perugini score for any cardiac amyloidosis (80% vs 99%) and ATTR subtypes (95% vs. 99%). Similarly to the visual scoring specificity of SPECT/CT SUVmax > 6.1 could be increased to 99%, if patients had no plasma cell dyscrasia.

**Table 3:** Sensitivity and specificity of Perugini score vs. clinical diagnosis planar radionuclide scan vs. clinically verified cardiac amyloidosis vs. SPECT/CT (n=136)

Planar radionuclide scan				SPECT/CT SUVmax >6.1
Any amyloidosis	Positive scan (Grade 1, 2 or 3), n	Negative scan (Grade 0), n	SEN and SPE (CI), %	SEN and SPE (Cl), %)
Cardiac amyloidosis	36	9	80 (0.66 to 0.89)	99 (0.93 to 1.00)
No cardiac amyloidosis	15	76	83 (0.75 to 0.90)	67 (0.54 to 0.79)

ATTR amyloidosis	Positive Scan (Grade 1, 2 or 3), n	Negative Scan (Grade 0), n	SEN and SPE (CI), %	SEN and SPE (Cl), %)
Cardiac ATTR amyloidosis	36	2	95 (0.83 to 0.99)	99 (0.94 to 1.00)
No cardiac ATTR amyloidosis	15	83	85 (0.76 to 0.91)	87 (0.73 to 0.94)

ATTR amyloidosis	Grade 2 or 3, n	Grade 0 or 1, n	SEN and SPE (CI), %	SEN and SPE (Cl), %)
Cardiac ATTR amyloidosis	33	5	87 (0.73 to 0.94)	84 (0.70 to 0.93)
No cardiac ATTR amyloidosis	0	98	100 (0.96 to 1.00)	100 (0.96 to 1.00)

Planar Radionuclide Scan and Absence of Clone vs. clinically verified cardiac ATTR amyloidosis vs. SPECT/CT (n=136)				
	Grade 2 or 3 + No Clone, n	Grade 0/1 or Clone, n	SEN and SPE (CI), %	SEN and SPE (Cl), %) SUVmax >6.1 / < 6.1
Cardiac ATTR amyloidosis	29	9	76 (0.61 to 0.87)	71 (0.56 to 0.83)
No cardiac ATTR amyloidosis	0	98	100 (0.96 to 1.00)	99 (0.94 to 1.00)*

Right column shows sensitivity and specificity when additionally stratified by cut-off SUVmax < 6.1. Cardiac amyloidosis was diagnosed based on EMB, biopsy, MRI, bone scan, monoclonal protein in urine/blood. *In one patient CA was ruled out (detailed in Supplemental Fig. 1). SEN, sensitivity; SPE, specificity; ATTR, transthyretin

**Figure 5:**
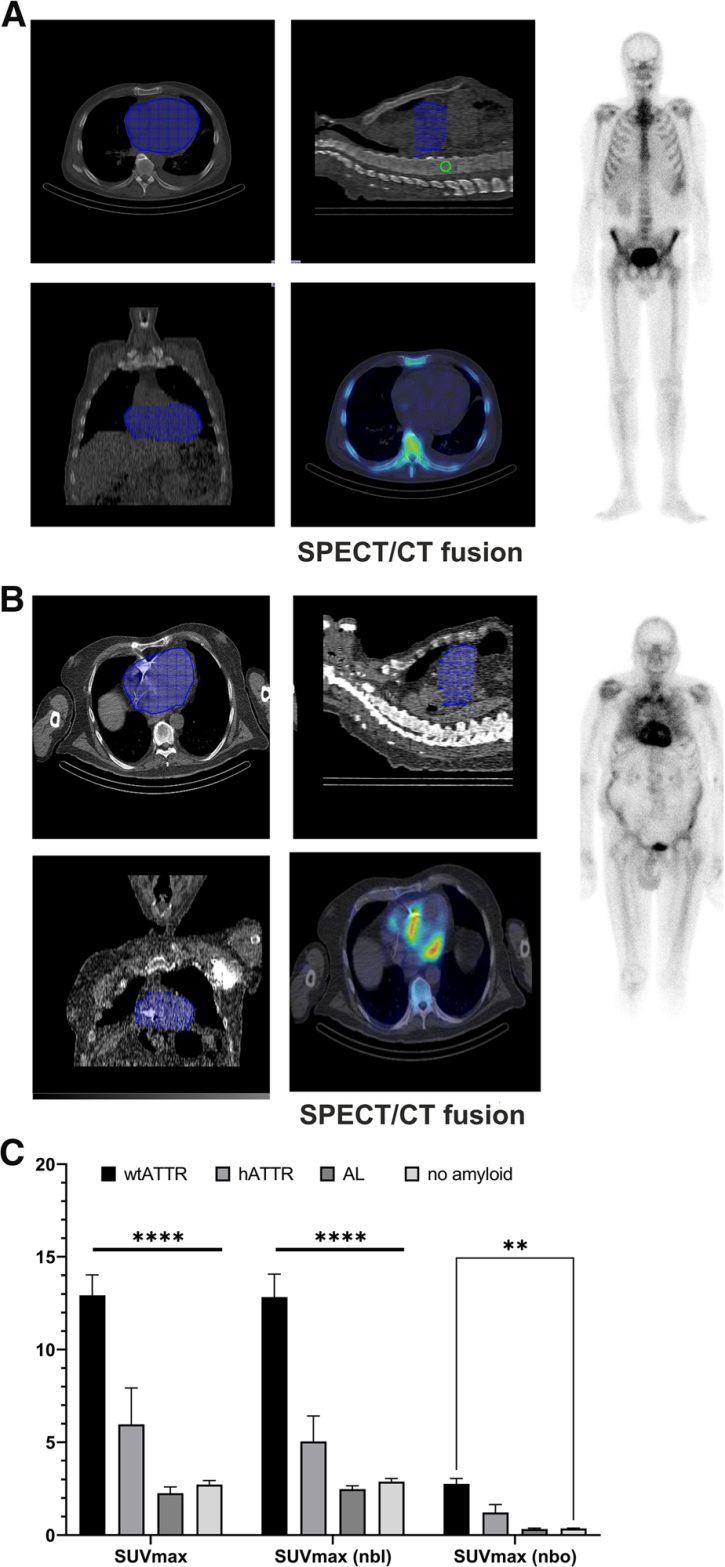
Representative example cases. VOI delineation in blue. (**A**) 66-year-old male with AL amyloidosis does not show any cardiac uptake in planar scintigraphy (Perugini 0) and in SPECT/CT images with a SUVmax of 1.6. (**B**) 84-year-old male with wtATTR amyloidosis and Perugini score 3 in the planar images with decreasing uptake in bone. SPECT/CT shows high tracer uptake in the left ventricular myocardium and a SUVmax of 26.7. (**C**) Difference in cardiac uptake of amyloidosis subtypes for the three SUV parameters. Patients with wildtype ATTR amyloidosis exhibited significantly higher uptake values compared (*p* < 0.001)

The delineation of the heart VOI in a low-dose CT is s shown in Figure 5A and B (blue area). In Figure 5A, a representative case of a patient with proven AL amyloidosis is demonstrated, showing a visual score of Perugini 0 in the planar images corresponding to a SUVmax of 1.57 in quantitative SPECT/CT. The second patient suffered from wtATTR amyloidosis and showed a highly pathological cardiac tracer uptake with decrease of tracer uptake in the bone (Perugini 3). SPECT/CT verifies high tracer uptake in the left ventricular myocardium with a SUVmax of 26.7 (Figure 5B). The mean SUV parameters for each amyloidosis subtype are in line with the characteristic uptake patterns, with the significantly higher uptake in wtATTR and hATTR compared to AL amyloidosis and patients without CA (*p* < 0.001) (Figure 5C). Overall, only one patient was reported as an outlier with a visual score of Perugini 1 and a relatively high SUVmax of 7.2 was measured (mean SUVmax in Perugini 1 group: 4.0 ± 0.46; mean SUVmax of patients without CA: 2.9 ± 0.17) (Supplemental Figure 1). Further no monoclonal protein or free light chains were found in urine and blood samples and eventually, a ‘non-amyloid’ cause of the HFpEF was diagnosed in this case.

## Discussion

In this study we propose a whole heart quantitative assessment of cardiac DPD tracer uptake in a large group of patients. Further, the diagnostic performance is comparable to the Perugini scoring system with partially higher sensitivity for the detection of CA.

Quantitative approaches may further increase the diagnostic value of bone scintigraphy for CA and have the potential to more thoroughly assess the burden of amyloid deposits, which is crucial to better risk-stratify patients with CA and may have a role in evaluation of response to anti-amyloid treatments.^10^ Currently SPECT and scintigraphy with bone-seeking tracers has not been shown to quantify response to therapies and therefore repeated scans are not clinically recommended.^19^ Yet this is based on semiquantitative ratios from planar images and not SPECT/CT quantification.

Visual scoring is a well-validated and relatively robust method; however, scoring depends on the readers’ experience to classify a patient according to the established 4-point system. Especially for patients with borderline cardiac uptake this score loses accuracy, when patients cannot be clearly rated as Perugini 1 or 2, for instance. In this case, the decision to perform an endomyocardial biopsy depends on the Perugini score. In the authors opinion, these cases might be relatively rare; however, the clinical consequence is immense, as the patient may have to undergo a procedure with a higher risk of complications. Due to that standardized uptake measurements are utterly important to allow comparability and repeatability of the results. Since Perugini et al. established the visual scoring many semiquantitative approaches from planar and SPECT images have been investigated in multiple ways. Most of the studies focused on variable ratios of cardiac uptake to a specified background uptake or to partial/whole body tracer uptake.^11,17,19–22^ Most studies indicate that bone scintigraphy and derived semiquantitative measurements provide reliable data, even for discrimination between AL and ATTR subtype.^23^ Most commonly the heart/to contralateral (H/CL) ROI approach is used, where a cut-off of ≥ 1.5 has been found to accurately differentiate ATTR-CA from AL-CA.^21^ A recent comparison of various semiquantitative indices proposed that a heart-to-whole-body ratio would be the method of choice in clinical settings.^17^

Most recently there have been endeavors for a more precise way to quantify cardiac tracer uptake from combined SPECT/CT data.^14,16^ A novel SPET/CT approach with heart segmentation compared to semiquantitative measurements in 74 patients with ATTR amyloidosis and different Perugini scores showed a good correlation between semiquantitative and quantitative measurements.^14^

Our data are in line with the current knowledge of SPECT/CT quantification in cardiac amyloidosis and other quantitative SPECT/CT approaches using bone-seeking tracers.^16,18,24–27^ Even data from a commercial quantitative SPECT/CT approach showed that the calculated uptake values in their cohort are comparable with those of our study.^16^ In that study patients with a Perugini score 3 had a mean SUVmax of 16.2 (*n *= 4). vs. 14.8 in our study. Another recent publication showed that SPECT/CT outperforms planar quantification methods and they provided data on a myocardial retention index, which could discriminate between higher Perugini grades.^18^ Despite the precise methodology, the quantitative calculations were based only on small volumes of interest, which were placed over various regions by using a commercially available quantification software. The authors did not elaborate on the systematics in VOI placement. In theory, a whole heart delineation yields the advantage of total quantification of cardiac tracer uptake, which could be robustly compared intra-individually in longitudinal studies. Further a sensitivity 100% and a specificity of 75% for the detection of any cardiac amyloidosis was reported, which is comparable to our data (sensitivity 99% / specificity 67%). Nonetheless, following the Gillmore Criteria, very high diagnostic specificity should be achieved to avoid false-positive cardiac ATTR amyloidosis in patients who actually have AL amyloidosis and therefore require chemotherapy.^3^ We showed that by combination of SUVmax cut-off > 6.1 and the absence of a detectable monoclonal protein the diagnostic specificity increases to 99%. Although this study focused on SUVmax as surrogate parameter, the whole heart quantification of cardiac amyloid deposits might be beneficial approach for patients undergoing consecutive scans, for example for measurement of therapy response, which is difficult and prone to observer variation for manual or threshold based approach as established in other studies.^16,18^

## New Knowledge Gained

To our knowledge this is the first study using a whole heart delineation for ^99m^Tc-DPD SPECT/CT quantification in large cohort with suspected cardiac amyloidosis. Whole heart SPECT/CT absolute quantification shows strong correlation with the visual Perugini score and high diagnostic performance with SUVmax cut-off >6.1. Specificity of our approach can be increased to 99% by ruling out elevated monoclonal proteins. This method is accessible and easily implementable at other centers.

## Supplementary Information

Below is the link to the electronic supplementary material.

Supplementary file1 (DOCX 203 kb)

Supplementary file2 (PPTX 105 kb)

Supplementary file3 (PPTX 2077 kb)

## Abbreviations

CA: Cardiac amyloidosis
AL: Light chain amyloidosis
(h/wt) ATTR: (Hereditary/wild type) transthyretin amyloidosis
EMB: Endomyocardial biopsy
^99m^Tc: ^99m^Technetium
SUV: Standardized uptake value
SPECT: Single photon emission computed tomography
CT: Computed tomography
VOI: Volume of interest
HFp(r)EF: Heart failure with preserved (reserved) ejection fraction
^99m^Tc-DPD: ^99m^Tc-3,3-diphosphono-1,2-Propanodicarboxylicacid
