# Supplementary material for: Quantitative 99mTc-DPD-SPECT/CT assessment of cardiac amyloidosis
Source: J Nucl Cardiol. 2022 May 13;30(1):101–11. doi: 10.1007/s12350-022-02960-3 (PMC9984322; doi:10.1007/s12350-022-02960-3)
Supplement: Supplementary file 1 — Supplementary file1 (DOCX 203 kb) [file 12350_2022_2960_MOESM1_ESM.docx]

# Supplementary Material

**Supplemental Tabl. 1.** Detailed diagnostic criteria of suspected cardiac amyloidosis.

| **Diagnosis of cardiac amyloidosis based on (N=45):** | **wtATTR, n (%)** | **hATTR, n (%)** | **AL, n (%)** |
| --- | --- | --- | --- |
| Endomyocardial biopsy | 17 (37.8%) | - | 1 (2.2 %) |
| extracardiac tissue biopsy + monoclonal protein / lightchain | - | - | 4 (8.9 %) |
| extracardiac tissue biopsy + Perugini >2 + absence of monoclonal protein | 1 (2.2 %) | - | - |
| extracardiac tissue biopsy + pathological imaging*  + absence of monoclonal protein | 3 (6.7 %) | 1 (2.2 %) | - |
| Perugini >2 + absence of monoclonal protein | 15 (33.3%) | 3 (6.7 %) | - |

wt/hATTR: wildtype/hereditary transthyretin amyloidosis, AL: lightchain amyloidosis. *pathological imaging in magnetic resonance imaging or echocardiography.


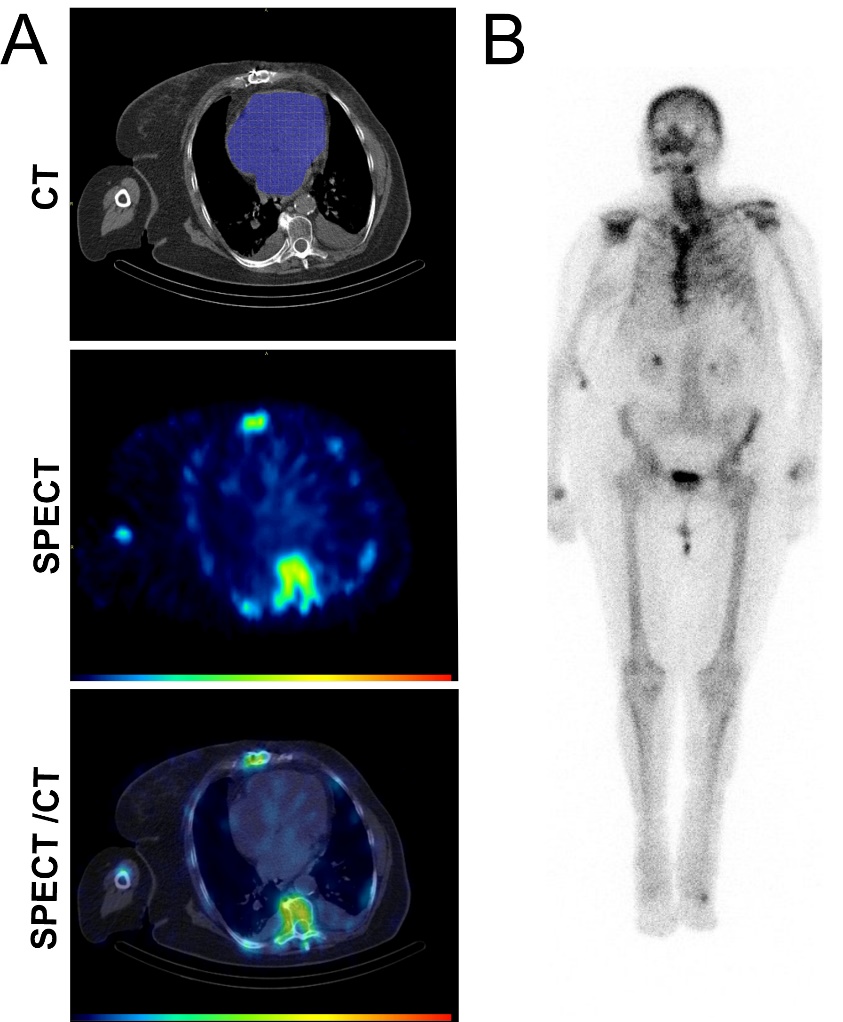


**Supplemental Fig. 1.** Female patient with visual score of Perugini 1 and higher than usual SUVmax of 7.2 but no reported cardiac amyloidosis. Visual interpretation of SPECT/CT images shows globally increased radiotracer uptake in the heart but not focused on myocardium.
