# Supplementary material for: Quantitative 99mTc-DPD-SPECT/CT assessment of cardiac amyloidosis
Source: J Nucl Cardiol. 2022 May 13;30(1):101–11. doi: 10.1007/s12350-022-02960-3 (PMC9984322; doi:10.1007/s12350-022-02960-3)
Supplement: Supplementary file 2 — Supplementary file2 (PPTX 105 kb) [file 12350_2022_2960_MOESM2_ESM.pptx]

## Slide 1
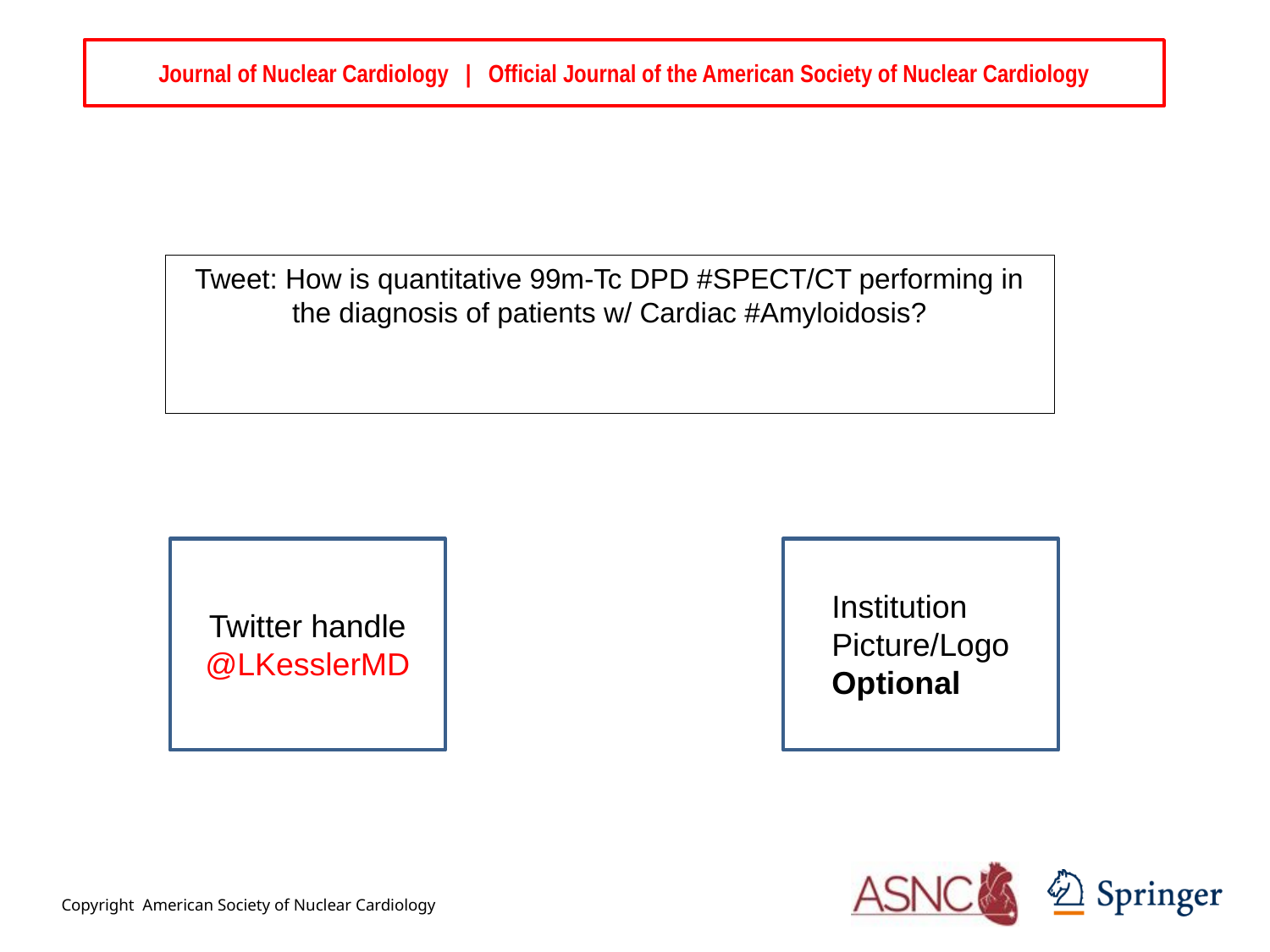

Journal of Nuclear Cardiology | Official Journal of the American Society of Nuclear Cardiology
Tweet: How is quantitative 99m-Tc DPD #SPECT/CT performing in the diagnosis of patients w/ Cardiac #Amyloidosis?
Twitter handle
@LKesslerMD
Institution
Picture/Logo
Optional
Copyright American Society of Nuclear Cardiology

## Slide 2
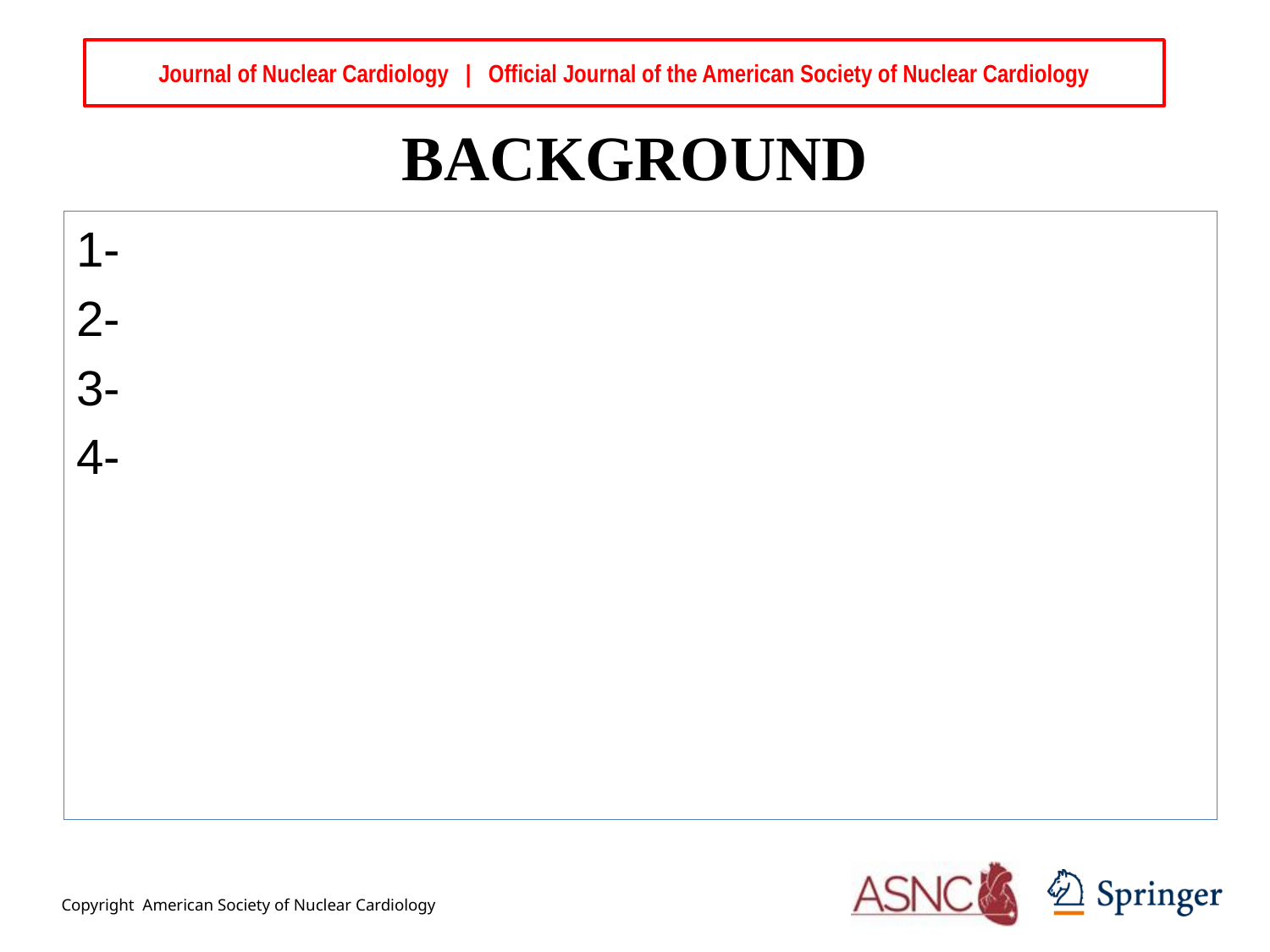

Journal of Nuclear Cardiology | Official Journal of the American Society of Nuclear Cardiology
# BACKGROUND
1-
2-
3-
4-
Copyright American Society of Nuclear Cardiology

## Slide 3
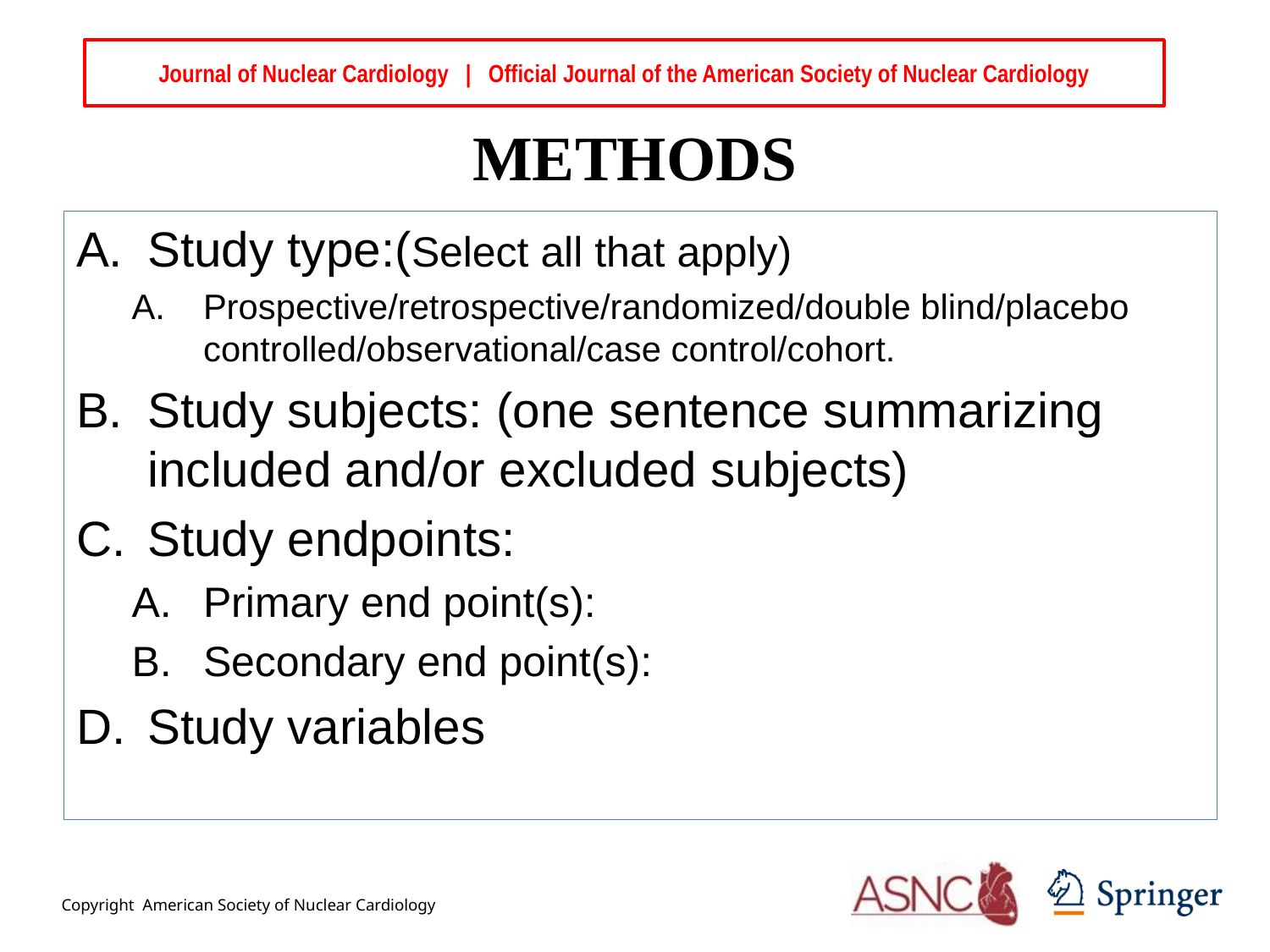

Journal of Nuclear Cardiology | Official Journal of the American Society of Nuclear Cardiology
# METHODS
Study type:(Select all that apply)
Prospective/retrospective/randomized/double blind/placebo controlled/observational/case control/cohort.
Study subjects: (one sentence summarizing included and/or excluded subjects)
Study endpoints:
Primary end point(s):
Secondary end point(s):
Study variables
Copyright American Society of Nuclear Cardiology

## Slide 4
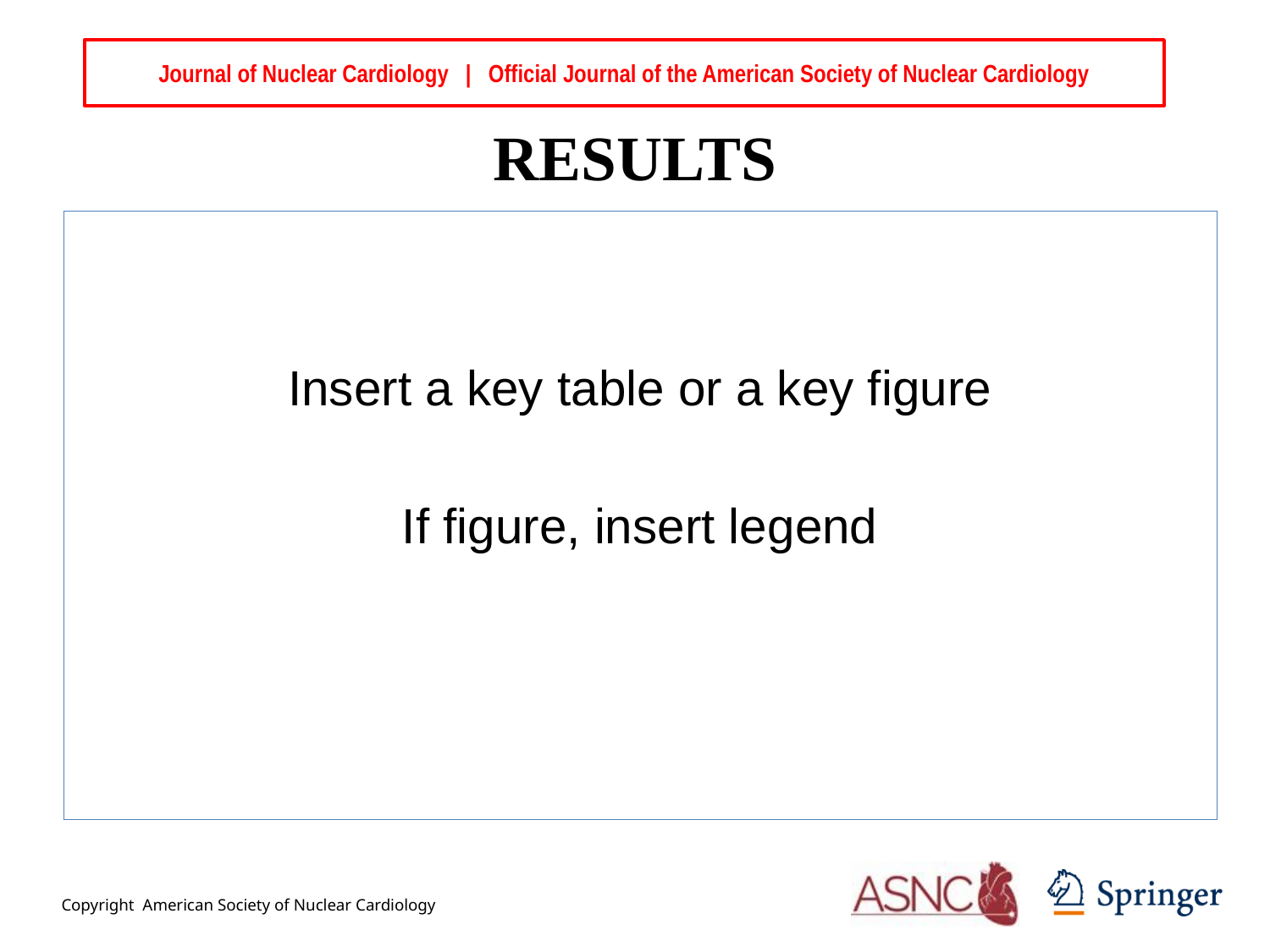

Journal of Nuclear Cardiology | Official Journal of the American Society of Nuclear Cardiology
# RESULTS
Insert a key table or a key figure
If figure, insert legend
Copyright American Society of Nuclear Cardiology

## Slide 5
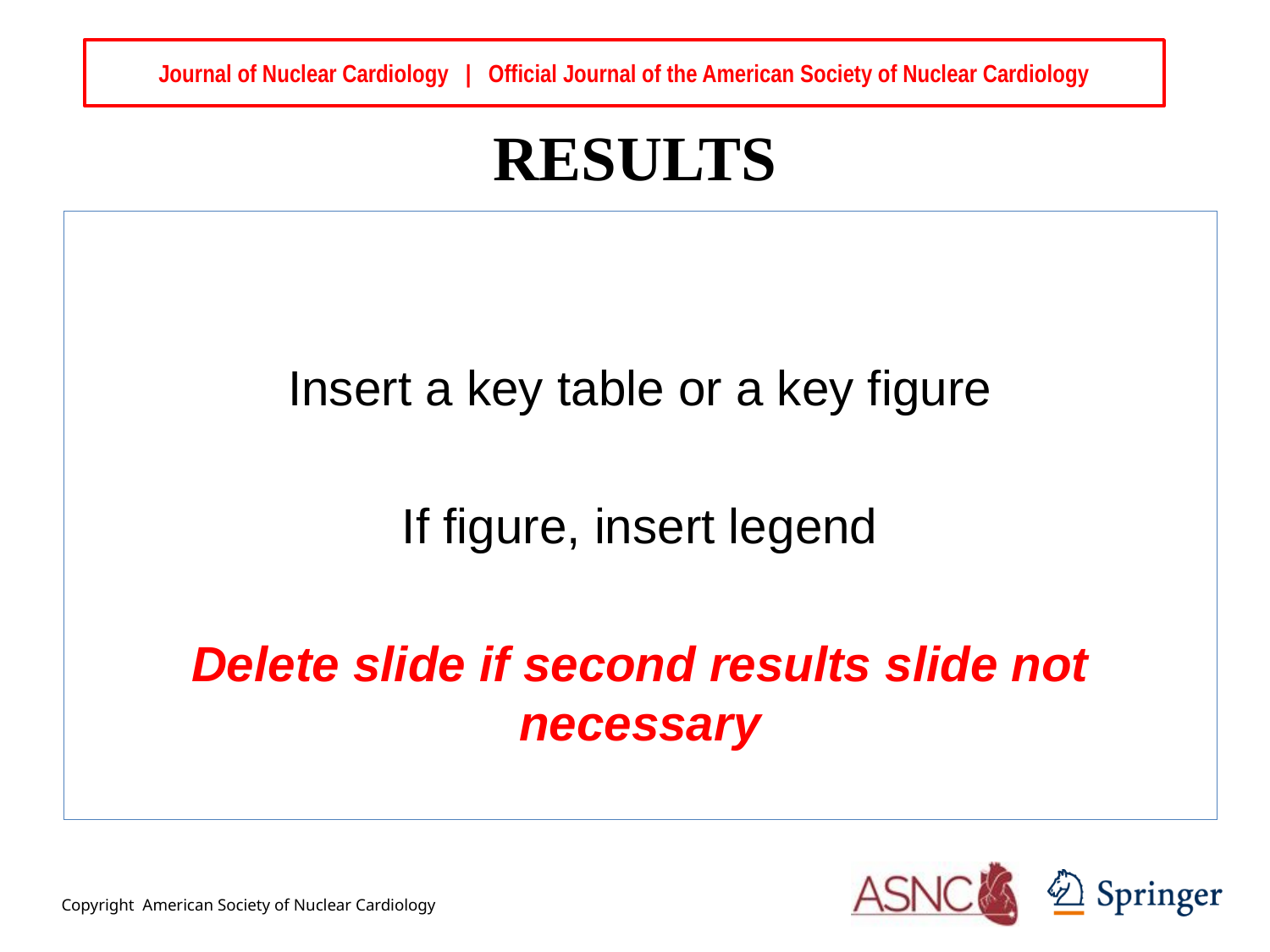

Journal of Nuclear Cardiology | Official Journal of the American Society of Nuclear Cardiology
# RESULTS
Insert a key table or a key figure
If figure, insert legend
Delete slide if second results slide not necessary
Copyright American Society of Nuclear Cardiology

## Slide 6
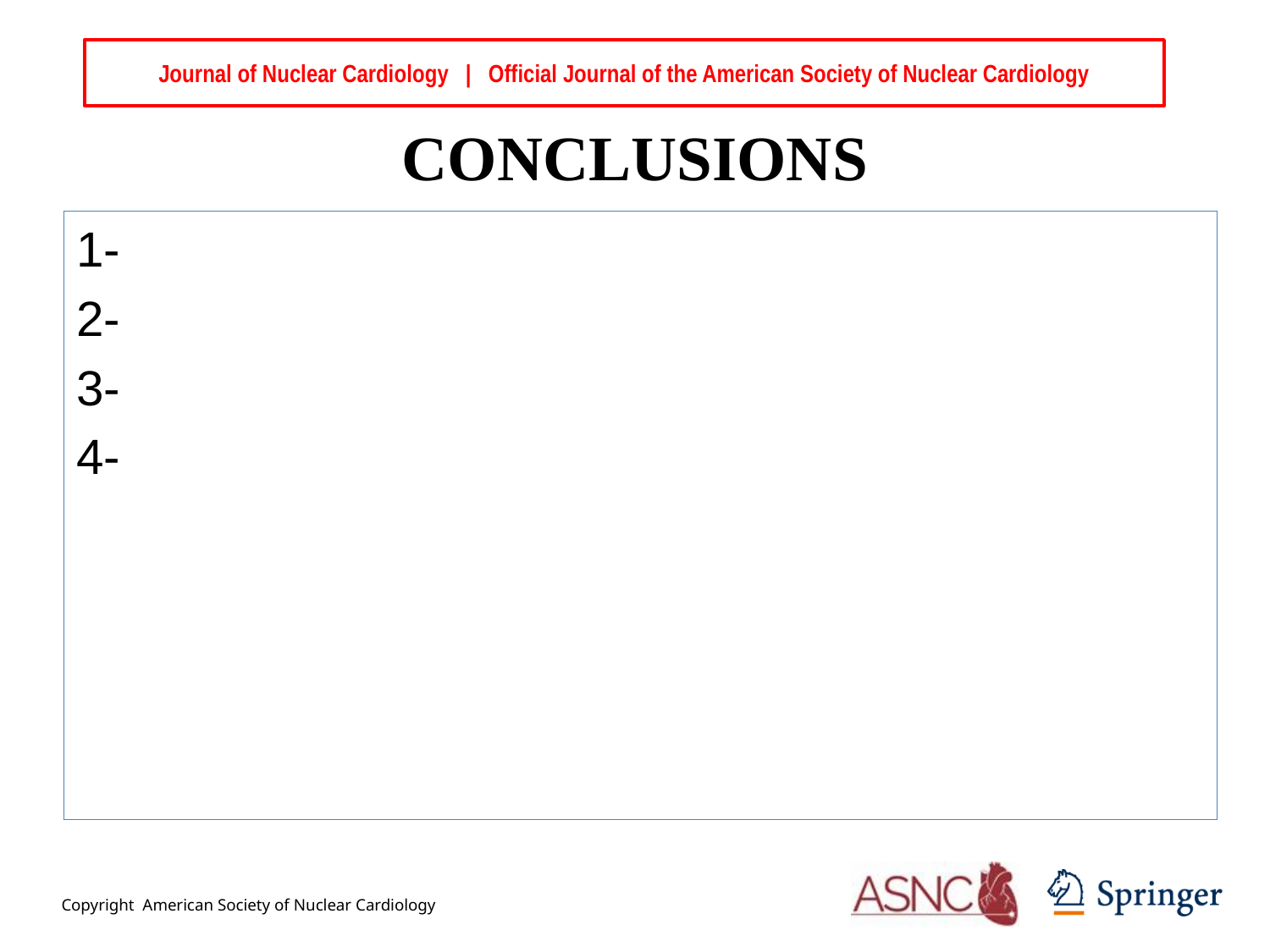

Journal of Nuclear Cardiology | Official Journal of the American Society of Nuclear Cardiology
# CONCLUSIONS
1-
2-
3-
4-
Copyright American Society of Nuclear Cardiology
