# Supplementary material for: Quantitative 99mTc-DPD-SPECT/CT assessment of cardiac amyloidosis
Source: J Nucl Cardiol. 2022 May 13;30(1):101–11. doi: 10.1007/s12350-022-02960-3 (PMC9984322; doi:10.1007/s12350-022-02960-3)
Supplement: Supplementary file 3 — Supplementary file3 (PPTX 2077 kb) [file 12350_2022_2960_MOESM3_ESM.pptx]

## Slide 1
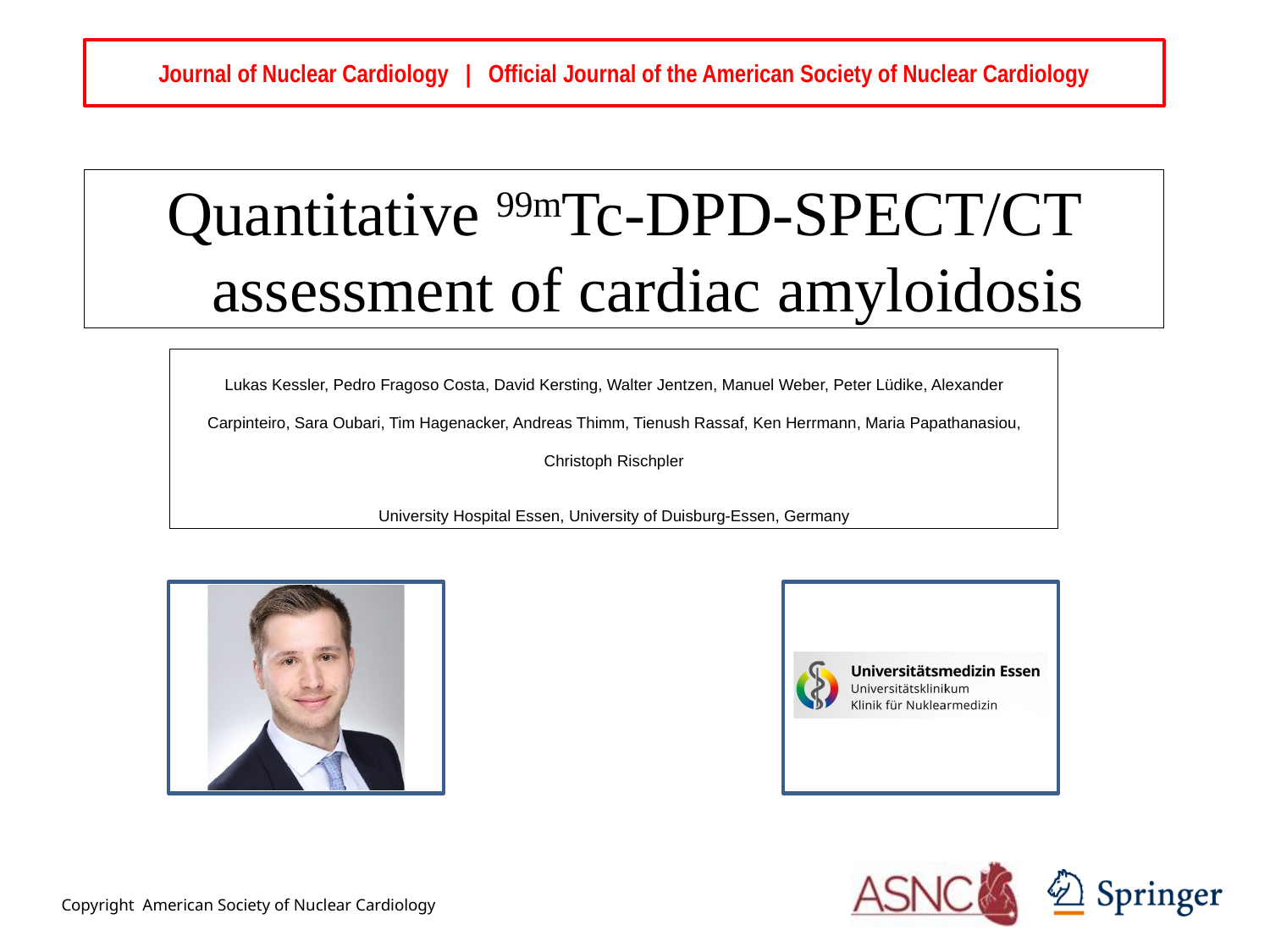

Journal of Nuclear Cardiology | Official Journal of the American Society of Nuclear Cardiology
# Quantitative 99mTc-DPD-SPECT/CT assessment of cardiac amyloidosis
Lukas Kessler, Pedro Fragoso Costa, David Kersting, Walter Jentzen, Manuel Weber, Peter Lüdike, Alexander Carpinteiro, Sara Oubari, Tim Hagenacker, Andreas Thimm, Tienush Rassaf, Ken Herrmann, Maria Papathanasiou, Christoph Rischpler
University Hospital Essen, University of Duisburg-Essen, Germany
Head shot of author
required
Copyright American Society of Nuclear Cardiology

## Slide 2
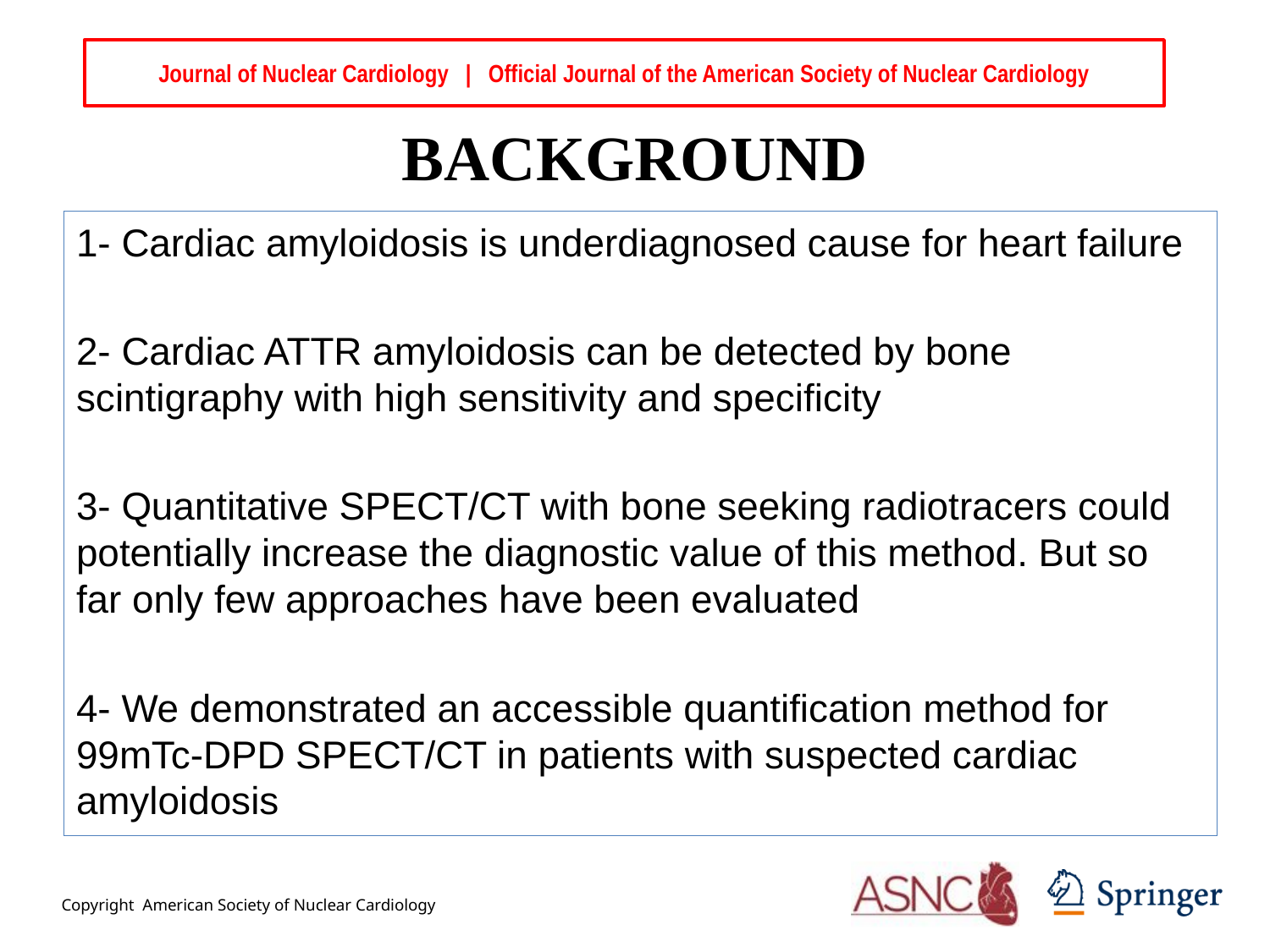

Journal of Nuclear Cardiology | Official Journal of the American Society of Nuclear Cardiology
# BACKGROUND
1- Cardiac amyloidosis is underdiagnosed cause for heart failure
2- Cardiac ATTR amyloidosis can be detected by bone scintigraphy with high sensitivity and specificity
3- Quantitative SPECT/CT with bone seeking radiotracers could potentially increase the diagnostic value of this method. But so far only few approaches have been evaluated
4- We demonstrated an accessible quantification method for 99mTc-DPD SPECT/CT in patients with suspected cardiac amyloidosis
Copyright American Society of Nuclear Cardiology

## Slide 3
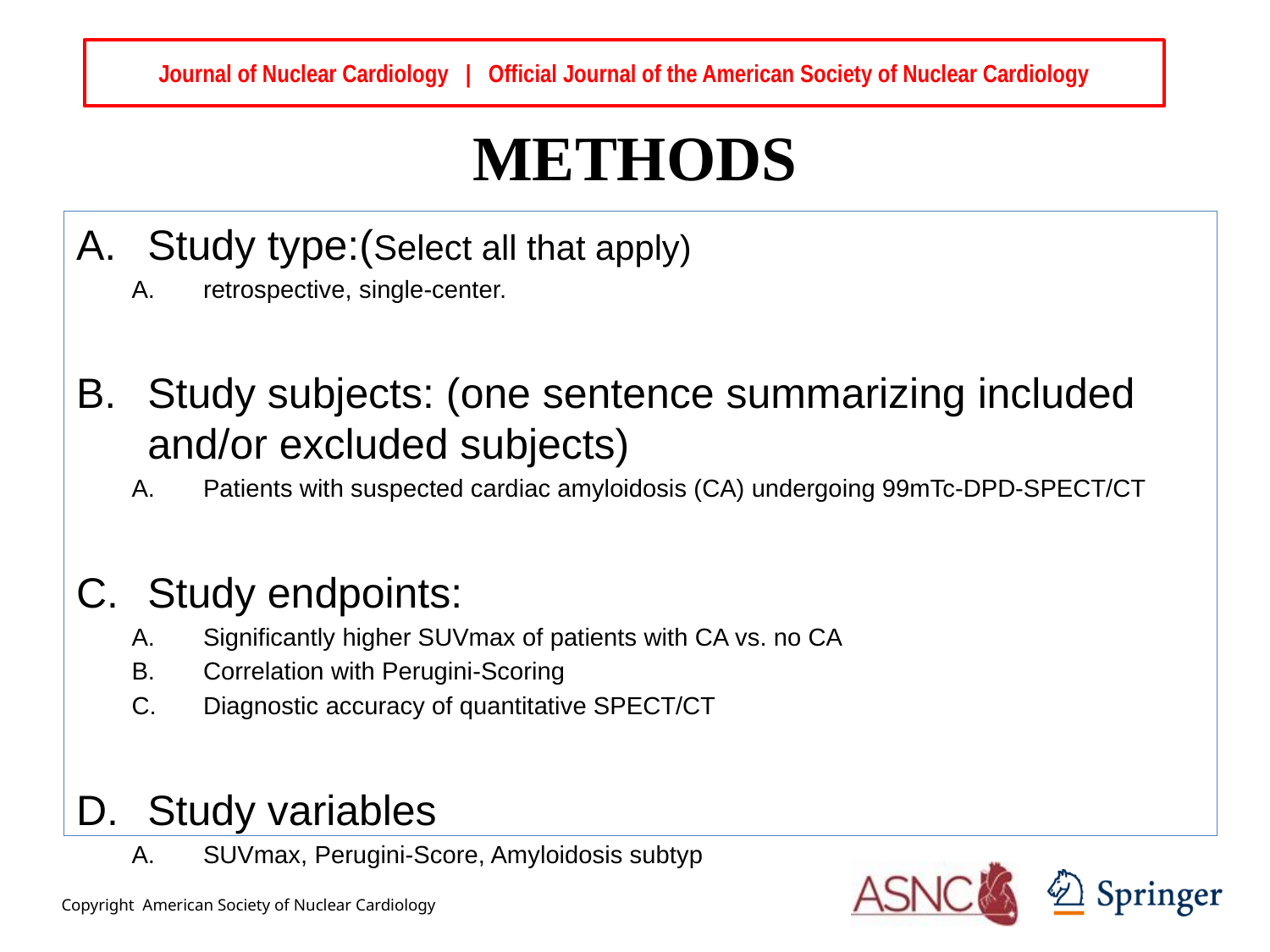

Journal of Nuclear Cardiology | Official Journal of the American Society of Nuclear Cardiology
# METHODS
Study type:(Select all that apply)
retrospective, single-center.
Study subjects: (one sentence summarizing included and/or excluded subjects)
Patients with suspected cardiac amyloidosis (CA) undergoing 99mTc-DPD-SPECT/CT
Study endpoints:
Significantly higher SUVmax of patients with CA vs. no CA
Correlation with Perugini-Scoring
Diagnostic accuracy of quantitative SPECT/CT
Study variables
SUVmax, Perugini-Score, Amyloidosis subtyp
Copyright American Society of Nuclear Cardiology

## Slide 4
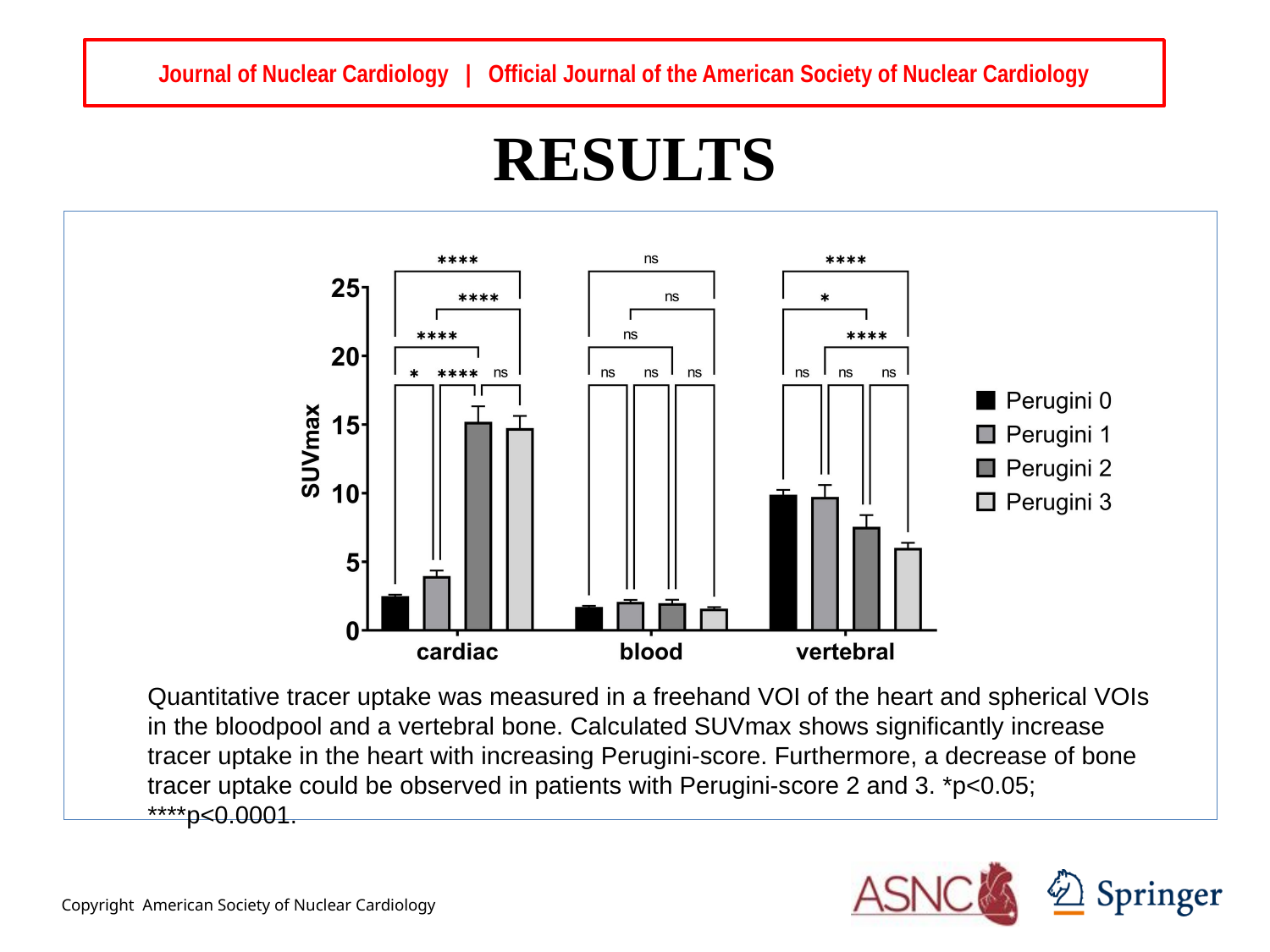

Journal of Nuclear Cardiology | Official Journal of the American Society of Nuclear Cardiology
# RESULTS
Insert a key table or a key figure
If figure, insert legend
Quantitative tracer uptake was measured in a freehand VOI of the heart and spherical VOIs in the bloodpool and a vertebral bone. Calculated SUVmax shows significantly increase tracer uptake in the heart with increasing Perugini-score. Furthermore, a decrease of bone tracer uptake could be observed in patients with Perugini-score 2 and 3. *p<0.05; ****p<0.0001.
Copyright American Society of Nuclear Cardiology

## Slide 5
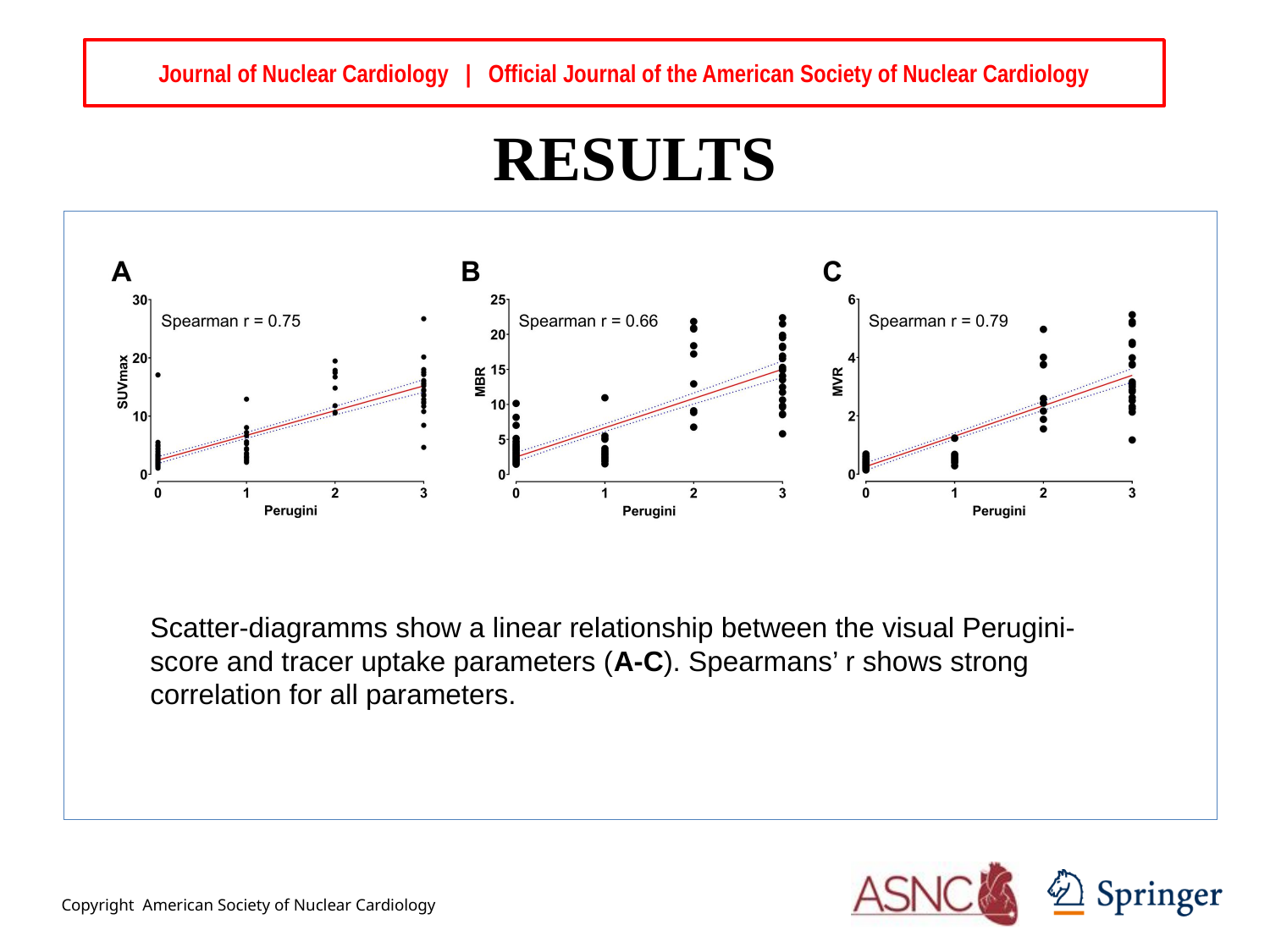

Journal of Nuclear Cardiology | Official Journal of the American Society of Nuclear Cardiology
# RESULTS
Insert a key table or a key figure
Scatter-diagramms show a linear relationship between the visual Perugini-score and tracer uptake parameters (A-C). Spearmans’ r shows strong correlation for all parameters.
Copyright American Society of Nuclear Cardiology

## Slide 6
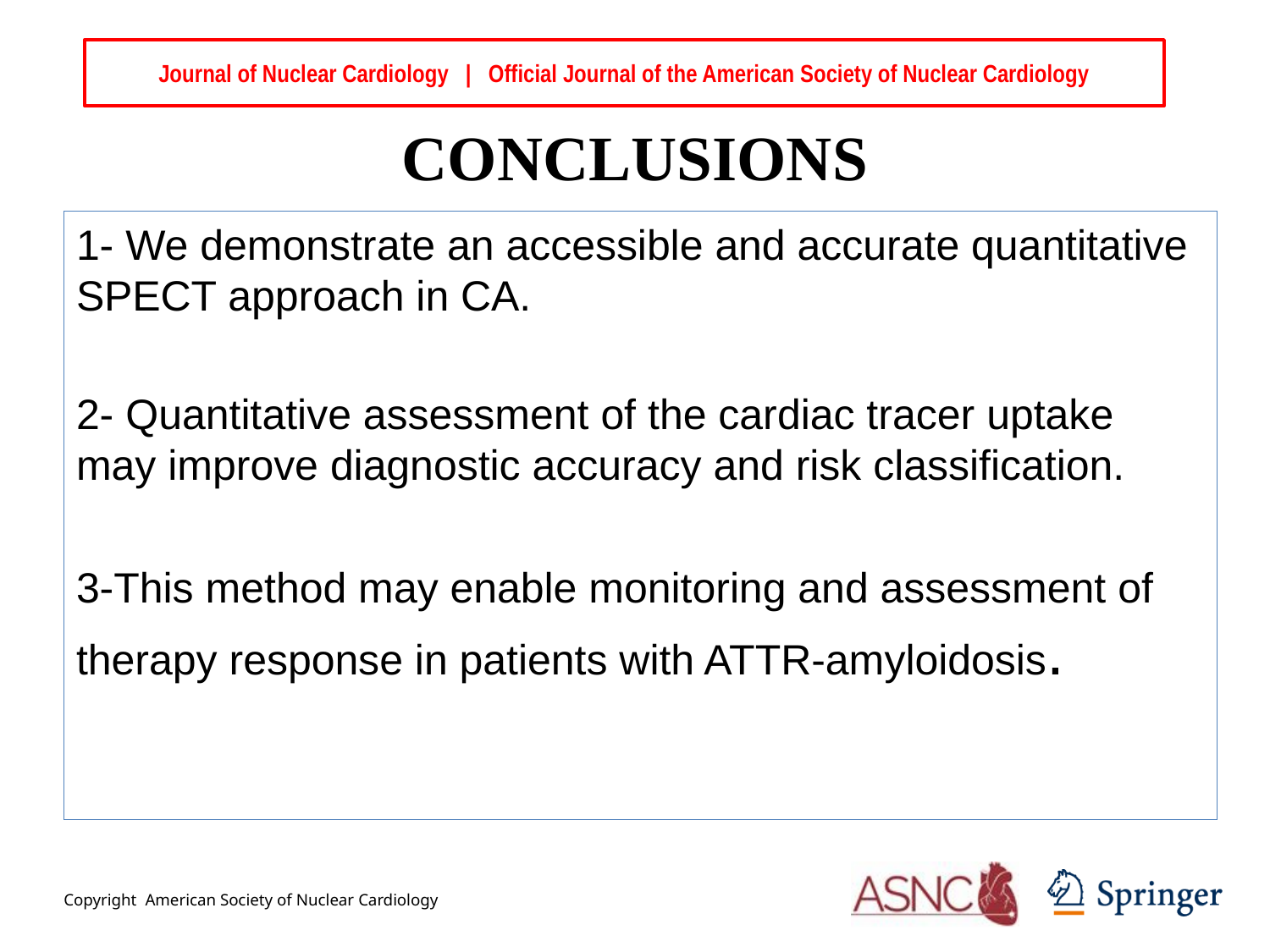

Journal of Nuclear Cardiology | Official Journal of the American Society of Nuclear Cardiology
# CONCLUSIONS
1- We demonstrate an accessible and accurate quantitative SPECT approach in CA.
2- Quantitative assessment of the cardiac tracer uptake may improve diagnostic accuracy and risk classification.
3-This method may enable monitoring and assessment of therapy response in patients with ATTR-amyloidosis.
Copyright American Society of Nuclear Cardiology
